# Association of Social and Geographic Vulnerability With In-Hospital Outcomes in Takotsubo Cardiomyopathy: Insights From a National Database

**DOI:** 10.7759/cureus.108638

**Published:** 2026-05-11

**Authors:** Teddy A Teddy, Edidiong Okon-Ben, Spencer Cadet, Abdelwahab Ahmed, Mustafa Marzoung, Siri Vummaneni, Kendall Bell

**Affiliations:** 1 Internal Medicine, Detroit Medical Center, Wayne State University, Detroit, USA; 2 Internal Medicine, HCA Healthcare, University of Central Florida Fort Walton Beach Hospital, Florida, USA; 3 Cardiology, Detroit Medical Center, Wayne State University, Detroit, USA

**Keywords:** acute cardiac care, cardiogenic shock, cardiovascular outcomes, geographic variation, healthcare access, rural health disparities in hospital mortality, socioeconomic status, takotsubo cardiomyopathy

## Abstract

Background

Takotsubo cardiomyopathy (TTC) is a transient, acute cardiac dysfunction syndrome triggered by emotional or physical stress. The pathophysiology involves catecholamine excess and microvascular dysfunction. The influence of geographic access to care and socioeconomic context on clinical outcomes remains poorly characterized. This study tested whether rural hospital setting and low community income level independently associate with adverse in-hospital outcomes among patients hospitalized with TTC.

Methodology

We conducted a retrospective cohort study using the 2022 National Inpatient Sample. Adult hospitalizations with a principal diagnosis of TTC were identified using International Classification of Diseases, Tenth Revision, Clinical Modification coding. Hospitalizations were stratified by hospital location and teaching status and by median community income quartile. The primary outcome was in-hospital mortality. Secondary outcomes included cardiogenic shock, acute respiratory failure, arrhythmia, invasive mechanical ventilation, length of stay, total hospital charges, and discharge disposition. Multivariable logistic regression was performed using STATA survey procedures to account for the complex survey design. Adjusted odds ratios (aORs) with 95% confidence intervals (CIs) were reported.

Results

A total of 4,738 hospitalizations met the inclusion criteria. This represented a weighted national estimate of 23,690 admissions. Rural hospitals accounted for 1,017 admissions or 21.5%. Patients from the lowest income quartile (Q1) accounted for 1,486 admissions or 31.4%. Rural hospitalizations showed higher crude in-hospital mortality compared with urban hospitalizations at 4.7% versus 2.8%, with a p-value less than 0.001. Patients from Q1 had higher mortality than those from the highest income quartile (Q4) at 4.3% versus 2.5%, with a p-value equal to 0.002. After multivariable adjustment using survey-weighted logistic regression, rural hospitalization remained independently associated with in-hospital mortality with an aOR of 1.58, 95% CI of 1.16 to 2.15, and p-value of 0.004. Low income status also remained independently associated with mortality, with an aOR of 1.41, 95% CI of 1.03 to 1.94, and p-value of 0.031.

Conclusions

In this nationally representative sample of patients hospitalized with TTC, rural hospital location and low socioeconomic status were independently associated with increased in-hospital mortality. These findings suggest that structural factors, including geographic access to specialized cardiovascular services and community-level economic resources, influence clinical outcomes in this condition.

## Introduction

Takotsubo cardiomyopathy (TTC) is a syndrome characterized by transient left ventricular systolic dysfunction that typically presents with clinical features resembling acute coronary syndrome [1]. The condition was first described in Japan in 1990 and derives its name from the characteristic apical ballooning pattern that resembles a takotsubo, a traditional octopus trap. Although initial reports suggested a relatively benign prognosis, subsequent larger studies have demonstrated meaningful rates of in-hospital complications, including cardiogenic shock (CS), arrhythmia, respiratory failure, and death, with mortality rates approximating 3 to 5% in contemporary cohorts. The pathophysiology of TTC remains incompletely understood, but the prevailing mechanistic model involves catecholamine-mediated myocardial stunning [2]. Emotional and physical stressors trigger a surge in circulating catecholamines, leading to direct myocyte injury, coronary microvascular dysfunction, and characteristic patterns of regional wall motion abnormality that extend beyond a single coronary artery distribution. For this reason, the condition is often conceptualized as a model of stress-induced cardiac injury, and it is plausible that chronic social stressors influence disease susceptibility and severity.

Rural populations in the United States face well-documented barriers to cardiovascular care. These include reduced access to cardiology specialty evaluation, longer travel distances to hospitals with cardiac catheterization capabilities, delayed transfer to tertiary centers for advanced interventions, and higher rates of uninsurance or underinsurance [3]. Rural hospitals themselves often lack on-site cardiac catheterization laboratories, invasive hemodynamic monitoring capabilities, and subspecialty critical care services. Consequently, rural patients with acute cardiovascular conditions experience delays in diagnosis, lower utilization of guideline-directed interventions, and worse clinical outcomes.

Similarly, psychological stress is common in low‑income communities, where sudden emotional distress can stun the heart muscle and mimic a heart attack even without blocked arteries. Beyond individual stress, the social environment in these communities often features weak social networks, high rates of neighborhood violence, racial or economic discrimination, and a lack of trusted community institutions. These chronic social stressors not only increase the baseline risk for TTC but also deprive residents of protective social support systems that might mitigate the physiological effects of acute emotional triggers. For individuals already facing food and housing insecurity, recurrent episodes of takotsubo can compound existing cardiovascular risks, leading to faster disease progression and worse outcomes in an already medically underserved population [4]. These factors contribute to both the development of TTC and the severity of its acute presentation. However, data specifically evaluating the interplay between hospital rurality, socioeconomic status, and TTC outcomes remain limited. Therefore, we examined the associations of rural hospital setting and community-level socioeconomic status with clinical outcomes among patients hospitalized with TTC using a nationally representative database [5]. We hypothesized that both rural hospital location and low community income would be independently associated with higher in-hospital mortality and greater complication rates after adjustment for patient demographic characteristics and clinical risk factors.

## Materials and methods

We performed a retrospective cross-sectional study using the 2022 National Inpatient Sample (NIS), which is the largest publicly available all-payer inpatient database in the United States [6]. The NIS is part of the Healthcare Cost and Utilization Project (HCUP) sponsored by the Agency for Healthcare Research and Quality (AHRQ) [5,7]. The database approximates a 20% stratified sample of discharges from community hospitals and includes approximately 7 million unweighted hospitalizations annually. When discharge weights are applied, the database permits the generation of nationally representative estimates of hospital utilization, outcomes, and costs. The sampling design incorporates stratification by hospital characteristics, including geographic region, bed size, teaching status, ownership, and urban-rural location, to ensure representativeness across diverse hospital types. Adult hospitalizations with a principal diagnosis of TTC were identified using the International Classification of Diseases, Tenth Revision, Clinical Modification (ICD-10-CM) code I51.81 [8]. This code specifically denotes stress-induced cardiomyopathy, Takotsubo syndrome, and transient left ventricular apical ballooning syndrome [9]. Hospitalizations were included if the patient was 18 years of age or older at the time of admission. We excluded hospitalizations with missing data on hospital location, patient demographic characteristics, or primary payer status, as these variables were necessary for exposure classification and covariate adjustment. To improve cohort specificity, we did not include hospitalizations with a secondary diagnosis of acute myocardial infarction as the principal diagnosis, as this could represent coding ambiguity between the two conditions.

The two primary exposure variables of interest were hospital location and socioeconomic status [9]. Hospital location was categorized as rural or urban based on the NIS designation, which classifies hospitals according to metropolitan statistical area (MSA) definitions from the United States Census Bureau.

Urban hospitals include those located in MSAs with populations exceeding 50,000, while rural hospitals include those in non-MSAs [5,7]. Socioeconomic status was defined using the median household income quartile for the patient’s residential ZIP code, as provided in the NIS data file. This variable is derived from ZIP code-level census data and is categorized into quartiles based on national distributions. For the primary comparative analysis, we focused on the lowest income quartile (Q1) and the highest income quartile (Q4) to maximize contrast in socioeconomic position. The middle two quartiles were included in descriptive analyses but were not the primary focus of comparative outcome assessment [10]. We included a comprehensive set of patient-level and hospital-level covariates to adjust for potential confounding. Patient-level covariates included age, sex, race and ethnicity, primary payer status, and Elixhauser comorbidity measures. Specific comorbid conditions of interest included hypertension, diabetes mellitus (DM), chronic kidney disease (CKD), obesity, tobacco use, anxiety disorders, depressive disorders, chronic lung disease, and coronary artery disease (CAD). These conditions were identified using ICD-10-CM diagnosis codes present in the secondary diagnosis fields. Hospital-level covariates included teaching status, geographic region, and hospital bed size category as defined by the NIS [11].

The primary outcome was in-hospital mortality, defined as death during the hospitalization period. Secondary outcomes included CS, acute respiratory failure (ARF), atrial fibrillation or flutter, ventricular arrhythmia, invasive mechanical ventilation (IMV), coronary angiography utilization, length of stay (LOS) in days, total hospital charges in United States dollars, and discharge disposition. Discharge disposition was categorized as discharge to home, including home with or without home health services, versus discharge to another facility, such as a skilled nursing facility, intermediate care facility, or other hospital. Secondary outcomes were identified using ICD-10-CM diagnosis codes for complications and procedure codes for interventions.

All statistical analyses were conducted using STATA version 17.0 (StataCorp, College Station, TX, USA) [8]. To account for the complex survey design of the NIS, all analyses incorporated survey weighting, stratification, and clustering using the svy prefix commands [9]. Specifically, we used the svyset command with the discharge weight variable, stratum identifier, and primary sampling unit identifier as specified in the NIS documentation. This approach ensures that point estimates, standard errors, and confidence intervals (CIs) are correctly calculated to reflect the sampling design and permit national extrapolation [11]. For descriptive analyses, categorical variables were summarized using weighted frequencies and percentages, while continuous variables were summarized using weighted means with standard deviations (SDs). We compared baseline characteristics and outcomes between hospital location groups and between income quartile groups using survey-adjusted chi-square tests for categorical variables, and survey-adjusted Wald tests for continuous variables. For multivariable analysis, we constructed a logistic regression model using the svy logit command to identify independent predictors of in-hospital mortality. Variables entered into the model were selected a priori based on clinical relevance and prior literature. These included age, sex, hospital rurality, income quartile, CKD, anxiety disorder, depression, DM, obesity, CAD, CS, and ARF. We included CS and ARF as covariates, recognizing that these are intermediate outcomes that lie on the causal pathway between the exposures and mortality. This approach allowed us to assess whether the effect of rurality and income persisted after accounting for these severe complications.

Model specification was assessed using the Hosmer-Lemeshow goodness-of-fit test adapted for survey data. Adjusted odds ratios (aORs) with 95% CIs were reported [7]. A two-sided p-value less than 0.05 was considered statistically significant for all hypothesis tests. We performed several sensitivity analyses to assess the robustness of our findings. First, we repeated the multivariable analysis excluding patients who were transferred from another hospital. Second, we conducted an analysis restricted to hospitals with cardiac catheterization capabilities [12,13]. Third, we examined the interaction between rurality and income.

## Results

A total of 4,738 unweighted hospitalizations met the inclusion criteria. This represented a weighted national estimate of 23,690 admissions for TTC in the United States during 2022. The mean age was 67.9 years with an SD of 12.4 years. Female patients accounted for 4,027 hospitalizations or 85.0% of the cohort. Overall, 1,486 hospitalizations occurred among patients from Q1 or 31.4% of admissions, while 882 hospitalizations were among patients from Q4 or 18.6% of admissions [8]. Rural hospitals accounted for 1,017 admissions or 21.5% of the cohort.

Comparison between urban and rural hospital settings revealed several notable differences (n = 4,738) (Table 1). Patients treated in rural hospitals were older on average, with a mean age of 70.3 years compared with 67.2 years for urban hospitals. This difference was statistically significant. Rural patients were more likely to be insured by Medicare, with 59.2% of rural hospitalizations covered by Medicare versus 51.1% of urban hospitalizations. The proportion of patients from Q1 was higher in rural hospitals at 37.3% compared with 29.8% in urban hospitals. Rural patients had a higher prevalence of CKD, DM, and tobacco use. Coronary angiography was performed in 52.1% of rural hospitalizations compared with 68.4% of urban hospitalizations. This was a difference of 16.3 percentage points.

**Table 1:** Baseline characteristics of the study population by hospital location. Comparison of 4,738 Takotsubo cardiomyopathy hospitalizations between urban (n = 3,721) and rural (n = 1,017) settings. Rural hospitals admitted older patients (70.3 vs. 67.2 years, p < 0.001), more Medicare beneficiaries (59.2% vs. 51.1%, p < 0.001), and a higher proportion of lowest income quartile patients (37.3% vs. 29.8%, p < 0.001). Rural patients had a greater prevalence of chronic kidney disease (22.5% vs. 17.1%, p < 0.001) and diabetes mellitus (26.1% vs. 22.9%, p = 0.041), yet received coronary angiography less frequently (52.1% vs. 68.4%, p < 0.001). Values are presented as n (weighted %) unless otherwise specified. Statistical tests: survey-adjusted Wald t-test for age (continuous variable); survey-adjusted Rao-Scott chi-square test (design-based F-statistic) for all categorical variables.

Variable	Urban (n = 3,721)	Rural (n = 1,017)	Test statistic	P-value
Age, mean ± SD	67.2 ± 12.1	70.3 ± 12.9	t = 7.24	<0.001
Female sex	3,196 (85.9)	831 (81.7)	F = 11.23	0.001
Medicare	1,902 (51.1)	602 (59.2)	F = 21.56	<0.001
Medicaid	572 (15.4)	119 (11.7)	F = 8.34	0.004
Lowest income quartile	1,107 (29.8)	379 (37.3)	F = 19.87	<0.001
Hypertension	2,617 (70.3)	747 (73.5)	F = 3.65	0.056
Diabetes mellitus	854 (22.9)	265 (26.1)	F = 4.18	0.041
Chronic kidney disease	635 (17.1)	229 (22.5)	F = 14.92	<0.001
Anxiety disorder	1,124 (30.2)	278 (27.3)	F = 3.03	0.082
Depression	982 (26.4)	251 (24.7)	F = 1.16	0.281
Tobacco use	826 (22.2)	263 (25.9)	F = 5.91	0.015
Coronary angiography	2,545 (68.4)	528 (52.1)	F = 84.36	<0.001

Comparison between Q1 and Q4 revealed significant differences in demographic and clinical profiles (n = 2,368) (Table 2). Patients from Q1 had a higher prevalence of CKD at 19.8% compared with 15.3% in Q4. Tobacco use was more common among low-income patients at 26.4% versus 18.9%. Anxiety disorders were also more prevalent in Q1 at 32.1% compared with 26.8%. Patients in Q1 were more likely to be insured by Medicaid, with coverage rates of 23.1% compared with 6.5% in Q4. Private insurance was more common in Q4 at 48.2% versus 26.3%. Racial and ethnic composition also differed significantly, with a higher proportion of Black and Hispanic patients in Q1.

**Table 2:** Socioeconomic profiling of 2,368 patients stratified by neighborhood income. The lowest quartile (Q1, n = 1,486) compared with the highest quartile (Q4, n = 882) revealed significant racial disparities, with Black patients comprising 18.6% of Q1 versus 5.0% of Q4 (p < 0.001). Medicaid coverage was nearly four times higher in Q1 (23.1% vs. 6.5%, p < 0.001), while private insurance dominated Q4 (48.2% vs. 26.3%, p < 0.001). Chronic kidney disease (19.8% vs. 15.3%, p = 0.005), tobacco use (26.4% vs. 18.9%, p < 0.001), and anxiety disorders (32.1% vs. 26.8%, p = 0.004) were all more prevalent in the lowest income quartile. Values are presented as n (weighted %) unless otherwise specified. Statistical tests: survey-adjusted Wald t-test for age (continuous variable); survey-adjusted Rao-Scott chi-square test (design-based F-statistic) for all categorical variables.

Variable	Lowest quartile (n = 1,486)	Highest quartile (n = 882)	Test statistic	P-value
Age, mean ± SD	66.8 ± 12.9	68.5 ± 11.8	t = 3.09	0.002
Female sex	1,256 (84.5)	758 (85.9)	F = 0.90	0.342
Race and ethnicity			F = 42.15	<0.001
White	1,024 (68.9)	712 (80.7)		
Black	276 (18.6)	44 (5.0)		
Hispanic	142 (9.6)	62 (7.0)		
Other	44 (3.0)	64 (7.3)		
Medicaid	343 (23.1)	57 (6.5)	F = 98.47	<0.001
Private insurance	391 (26.3)	425 (48.2)	F = 97.23	<0.001
Medicare	729 (49.1)	389 (44.1)	F = 5.61	0.018
Hypertension	1,058 (71.2)	618 (70.1)	F = 0.34	0.562
Diabetes mellitus	358 (24.1)	201 (22.8)	F = 0.54	0.462
Chronic kidney disease	294 (19.8)	135 (15.3)	F = 7.85	0.005
Obesity	287 (19.3)	186 (21.1)	F = 1.16	0.281
Tobacco use	392 (26.4)	167 (18.9)	F = 16.29	<0.001
Anxiety disorder	477 (32.1)	236 (26.8)	F = 8.35	0.004
Depression	399 (26.9)	221 (25.1)	F = 0.98	0.322
Chronic lung disease	248 (16.7)	132 (15.0)	F = 1.26	0.261
Coronary artery disease	327 (22.0)	186 (21.1)	F = 0.29	0.591

Crude in-hospital mortality was significantly higher in rural hospitals compared with urban hospitals (n = 4,738) (Table 3). Among rural hospitalizations, 4.7% resulted in death. Among urban hospitalizations, 2.8% resulted in death. This was an absolute difference of 1.9 percentage points. Rural hospitalizations also had higher rates of CS at 9.1% versus 7.4%, ARF at 18.6% versus 13.2%, and IMV at 5.7% versus 3.8%. All of these differences were statistically significant. Mean LOS was longer in rural hospitals, with a mean of 6.2 days compared with 5.1 days in urban hospitals. Despite the higher severity of illness, rural patients had lower rates of coronary angiography and were less frequently discharged home. Discharge to home occurred in 61.8% of rural hospitalizations compared with 71.5% of urban hospitalizations. Total hospital charges were lower in rural hospitals, with mean charges of $54,910 compared with $61,840 in urban hospitals.

**Table 3:** In-hospital outcomes by hospital location. This table compares clinical outcomes between urban (n = 3,721) and rural (n = 1,017) hospitals. Rural hospitals had higher mortality (4.7% vs. 2.8%, p < 0.001), more cardiogenic shock (9.1% vs. 7.4%, p = 0.048), more respiratory failure (18.6% vs. 13.2%, p < 0.001), longer stays (6.2 vs. 5.1 days), and lower discharge to home (61.8% vs. 71.5%, p < 0.001). Values are presented as n (weighted %) unless otherwise specified. Statistical tests: survey-adjusted Wald t-test for length of stay and hospital charges (continuous variables); survey-adjusted Rao-Scott chi-square test (design-based F-statistic) for all categorical outcomes.

Outcome	Urban (n = 3,721)	Rural (n = 1,017)	Test statistic	P-value
In-hospital mortality	103 (2.8)	48 (4.7)	F = 12.84	<0.001
Cardiogenic shock	276 (7.4)	93 (9.1)	F = 3.92	0.048
Acute respiratory failure	491 (13.2)	189 (18.6)	F = 19.45	<0.001
Atrial fibrillation or flutter	764 (20.5)	214 (21.0)	F = 0.11	0.739
Ventricular arrhythmia	118 (3.2)	42 (4.1)	F = 2.22	0.137
Invasive mechanical ventilation	143 (3.8)	58 (5.7)	F = 7.58	0.006
Coronary angiography	2,545 (68.4)	528 (52.1)	F = 84.36	<0.001
Discharge to home	2,661 (71.5)	628 (61.8)	F = 32.41	<0.001
Length of stay, days, mean ± SD	5.1 ± 3.7	6.2 ± 4.3	t = 7.89	<0.001
Total hospital charges, USD, mean ± SD	61,840 ± 29,220	54,910 ± 26,470	t = 6.54	<0.001

Patients from Q1 had worse outcomes than those from Q4 across multiple measures (n = 2,368) (Table 4). In-hospital mortality was 4.3% in Q1 compared with 2.5% in Q4. This was an absolute difference of 1.8 percentage points. CS occurred in 9.2% of low-income patients versus 6.1% of high-income patients. ARF rates were 16.5% and 11.2%, respectively. IMV was required in 4.8% of low-income patients compared with 2.7% of high-income patients. LOS was longer for patients in Q1, with a mean of 5.9 days compared with 4.8 days for those in Q4. Discharge to home was less common among low-income patients, occurring in 65.4% of cases versus 73.8% of high-income cases. Coronary angiography utilization was also lower in Q1 at 60.3% compared with 70.0% in Q4.

**Table 4:** Outcomes by income quartile (n = 2,368). Comparison of in‑hospital outcomes between Takotsubo cardiomyopathy patients from the lowest (Q1, n = 1,486) and highest (Q4, n = 882) median community income quartiles. Categorical variables are presented as weighted frequencies and percentages; continuous variables are presented as weighted means ± standard deviations (SD). Mean length of stay was 1.1 days longer for Q1 patients (5.9 vs. 4.8 days, p < 0.001). Total hospital charges were paradoxically lower in Q1 patients ($58,740 vs. $63,920, p < 0.001), despite their longer hospital stay and higher complication rates. All p-values are two‑sided, and p < 0.05 was considered statistically significant. Statistical tests used: survey‑adjusted chi‑square tests for categorical outcomes (χ² values shown) and survey‑adjusted Wald tests for continuous outcomes (t-values shown).

Outcome	Lowest quartile (Q1) (n = 1,486)	Highest quartile (Q4) (n = 882)	Test statistic	P-value
In-hospital mortality, n (%)	64 (4.3)	22 (2.5)	χ² = 9.58	0.002
Cardiogenic shock, n (%)	137 (9.2)	54 (6.1)	χ² = 8.32	0.004
Acute respiratory failure, n (%)	245 (16.5)	99 (11.2)	χ² = 12.91	<0.001
Invasive mechanical ventilation, n (%)	72 (4.8)	24 (2.7)	χ² = 6.82	0.009
Coronary angiography, n (%)	896 (60.3)	617 (70.0)	χ² = 5.34	0.021
Discharge to home, n (%)	972 (65.4)	651 (73.8)	χ² = 4.68	0.031
Length of stay, days, mean ± SD	5.9 ± 4.0	4.8 ± 3.3	t = 7.21	<0.001
Total hospital charges, USD, mean ± SD	58,740 ± 27,610	63,920 ± 30,480	t = 4.19	<0.001

**Figure 1:**
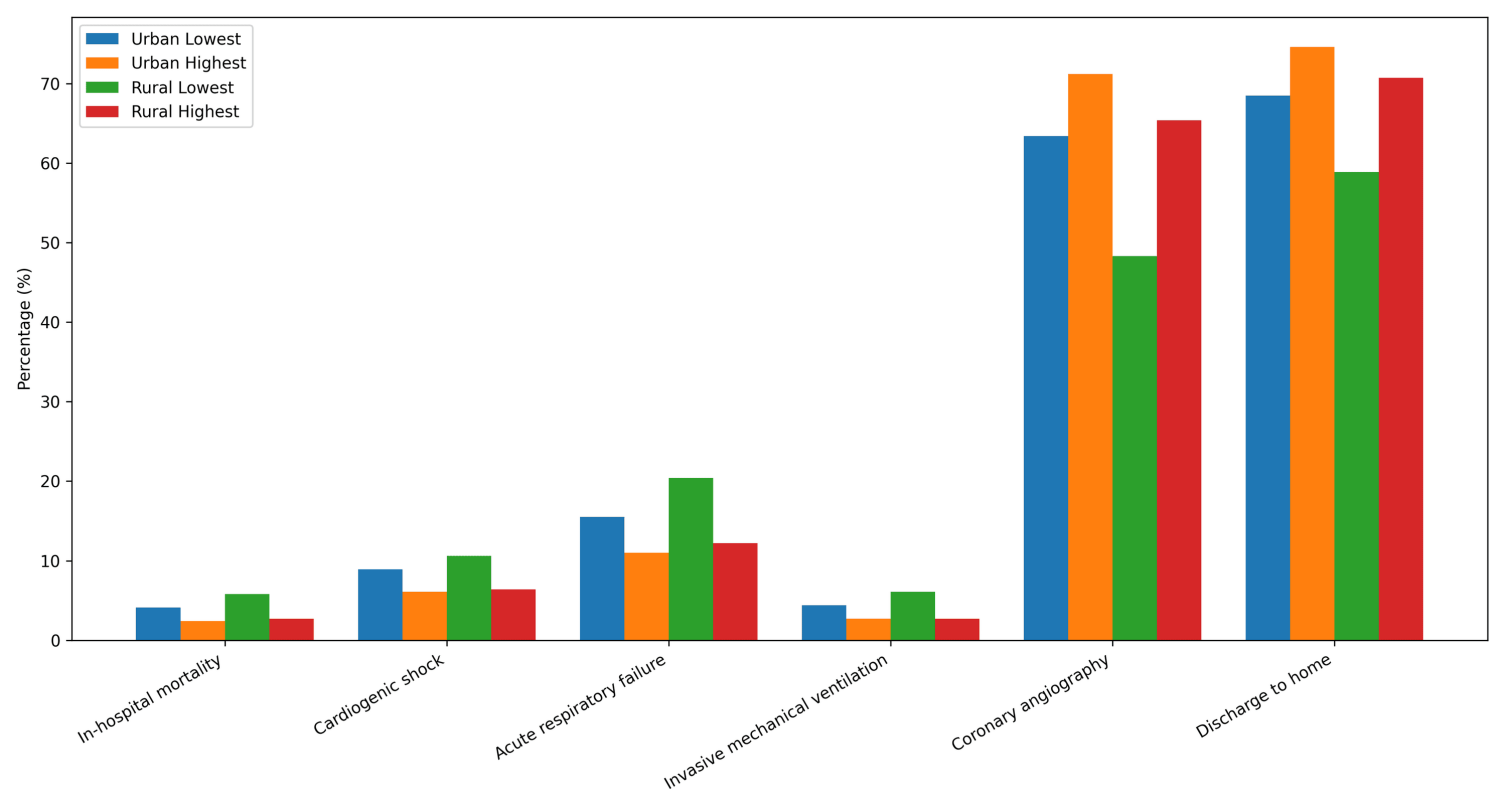
Outcomes by combined hospital location and income quartile. Combined effect of geographic and economic vulnerability across four patient subgroups (total n = 2,368). The rural lowest income subgroup (n = 379) exhibited the worst outcomes: mortality of 5.8% versus 2.4% in the urban highest income subgroup (n = 694). Cardiogenic shock occurred in 10.6% of rural low-income patients compared with 6.1% of urban high-income patients. Coronary angiography utilization ranged from 48.3% in the rural lowest subgroup to 71.2% in the urban highest subgroup. Discharge to home showed a similar gradient, from 58.9% in the rural lowest to 74.6% in the urban highest subgroup. The rural highest income subgroup included 421 patients, and the urban lowest income subgroup included 874 patients.

Patients in the rural Q1 subgroup had the highest in-hospital mortality at 5.8% compared with 2.4% in the urban Q4 subgroup (n = 2,368) (Figure 1). The rural Q1 group also had the highest rates of CS at 10.6% and ARF at 20.4%. Coronary angiography rates were lowest in the rural Q1 group at 48.3%. The urban Q4 group had the highest angiography rate at 71.2%. Discharge to home was lowest in the rural Q1 group at 58.9% and highest in the urban Q4 group at 74.6%.

**Figure 2:**
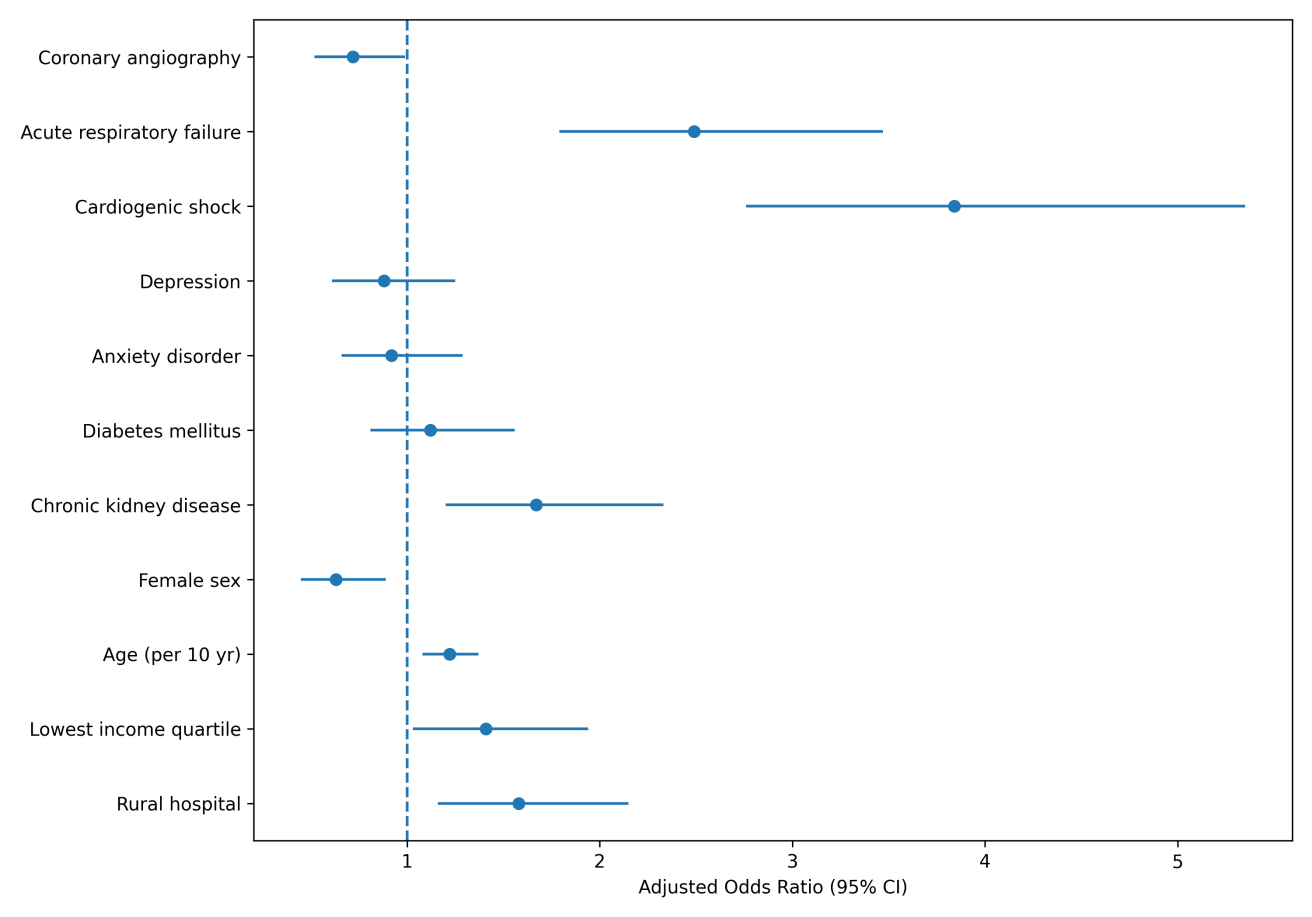
Multivariable logistic regression for in-hospital mortality. Independent predictors of in hospital mortality from survey weighted multivariable logistic regression (n = 4,738). Rural hospital location independently increased mortality odds by 58% (adjusted odds ratio (aOR) = 1.58, 95% confidence interval (CI) = 1.16 to 2.15, p = 0.004). Lowest income quartile status conferred a 41% increase in mortality odds (aOR = 1.41, 95% CI = 1.03 to 1.94, p = 0.031). Cardiogenic shock (aOR = 3.84, p < 0.001) and acute respiratory failure (aOR = 2.49, p < 0.001) were the strongest mortality predictors. Female sex (aOR = 0.63, p = 0.009) and coronary angiography (aOR = 0.72, p = 0.046) were independently associated with reduced mortality.

Rural hospitalization remained independently associated with in-hospital mortality after adjustment for patient demographic characteristics, comorbidities, and complications (n = 4,738) (Figure 2). The aOR for rural hospital location was 1.58 with a 95% CI of 1.16 to 2.15. This indicated a 58% increase in the odds of mortality for patients treated in rural hospitals compared with urban hospitals. Q1 also remained independently associated with in-hospital mortality. The aOR for low income status was 1.41 with a 95% CI of 1.03 to 1.94. This indicated a 41% increase in the odds of mortality for patients from Q1 compared with those from Q4. Additional independent predictors of in-hospital mortality included age, with a per-decade increase associated with a 22% increase in mortality odds. CKD was associated with a 67% increase in mortality odds. CS and ARF were strongly associated with mortality, with aOR of 3.84 and 2.49, respectively. Female sex was associated with lower mortality, with an aOR of 0.63. Coronary angiography was associated with reduced mortality odds, with an aOR of 0.72. The CI narrowly excluded the null.

Sensitivity analyses yielded consistent results [8]. When the analysis was restricted to patients not transferred from another hospital, the aOR for rural mortality was 1.54 with a 95% CI of 1.09 to 2.18. This was similar to the primary analysis. When the analysis was limited to hospitals with cardiac catheterization capabilities, the rural mortality difference was attenuated but remained elevated, with an aOR of 1.36 and a CI that crossed the null. The interaction term between rurality and income was not statistically significant.

## Discussion

Our finding that rural hospital location independently predicted higher in-hospital mortality among patients with TTC extends prior work on geographic disparities in cardiovascular outcomes. Several structural factors likely contribute to this association. Rural hospitals have lower rates of on-site cardiac catheterization [14]. They also have less experience with the management of CS and other complications that arise in TTC, given the lower volume of such cases. Third, transfer protocols to tertiary centers are slower or less well established in rural settings. This leads to delays in escalation of care when complications develop [15]. Beyond these healthcare system barriers, the chronic psychological and socioeconomic stress prevalent in rural and low‑income communities may directly prime the sympathetic nervous system. Persistent activation due to financial strain, housing insecurity, and social isolation can lead to baseline catecholamine excess, downregulation of adrenergic receptors, and enhanced microvascular reactivity. Consequently, when an acute trigger occurs, the myocardium is primed for a more severe TTC response, including larger areas of stunning and a higher likelihood of cardiogenic shock or death. This pathophysiological priming, an example of allostatic load, bridges the statistical association between rurality and mortality with the underlying biology of stress‑induced cardiomyopathy.

The independent association between low community income and adverse outcomes in TTC highlights the role of socioeconomic factors in this stress-mediated condition [16]. Several plausible pathways link socioeconomic disadvantage to worse outcomes [17]. Chronic exposure to social and economic stressors leads to heightened sympathetic tone and increased vulnerability to acute stress-induced myocardial injury. This chronic stress burden can be conceptualized as allostatic load. Allostatic load is the cumulative wear and tear on physiological systems resulting from repeated adaptation to stressors. Individuals in low-income communities also face barriers to accessing primary care. This results in delayed diagnosis of comorbid conditions and poorer baseline health status at the time of acute hospitalization. Health literacy, social support networks, and post-discharge resources differ systematically by income level. These factors influence both the acute course of illness and recovery trajectory [18].

The lower utilization of coronary angiography among both rural patients and low-income patients is a finding with important clinical implications [19]. Coronary angiography serves both diagnostic and prognostic purposes in TTC. It confirms the absence of obstructive coronary disease. It establishes the characteristic wall motion abnormality pattern. It rules out acute coronary syndrome as an alternative diagnosis [19]. Lower angiography rates reflect resource constraints in rural hospitals, differences in practice patterns, or a higher proportion of patients deemed too unstable for transfer or procedure [20]. The association we observed between angiography and reduced mortality in the multivariable model, though of borderline statistical significance, suggests that efforts to improve diagnostic evaluation in underserved settings could improve outcomes [21].

From a methodological perspective, the use of survey-weighted analysis in STATA was essential for producing valid national estimates from the NIS [22]. The svy prefix commands appropriately account for the stratified sampling design, which oversamples certain hospital types to improve representativeness. Failure to apply these survey weights would result in underestimated standard errors and potentially spurious statistical significance. Our approach of using svy logit with the appropriate weighting, stratification, and clustering variables ensures that the reported CI and p-values accurately reflect the sampling variability inherent in the database [13].

Several limitations of this study warrant detailed consideration. The NIS is an administrative database originally designed for health services research rather than clinical research. As such, it is subject to limitations inherent to administrative data [16]. Coding errors, including undercoding or overcoding of diagnoses and procedures, occur. The diagnosis of TTC was identified using the ICD-10-CM code I51.81. This code has not been validated against clinical criteria such as the International Takotsubo Diagnostic Criteria or the Mayo Clinic criteria [18]. While this code is specific for the condition, we cannot verify that all cases met established diagnostic criteria using angiographic, echocardiographic, or cardiac MRI data [19]. Misclassification of acute myocardial infarction as TTC, or vice versa, could bias our estimates. Such misclassification would likely be non-differential with respect to the exposures of interest. The NIS is a discharge-level database rather than a patient-level database [21]. If a patient was transferred from one hospital to another during the course of their illness, the transfer could be captured as two separate discharge records [22]. Our primary analysis did not link transfer records. This potentially led to double-counting of complex cases and incomplete capture of the full episode of care. To address this limitation, we conducted a sensitivity analysis excluding patients who were transferred from another hospital. This yielded consistent results. However, we cannot definitively account for all transfers or ensure that the full clinical trajectory is accurately represented.

The socioeconomic status variable in the NIS is based on median household income for the patient’s ZIP code rather than individual or household income [14]. This ecological measure misclassifies some individuals, particularly those whose personal income diverges substantially from the median of their neighborhood. Such misclassification would likely bias our estimates toward the null. This means that the true association between individual-level income and outcomes may be stronger than we observed. The NIS lacks direct measures of several factors that may be important confounders in the relationship between rurality, income, and outcomes. These include psychological stress, social support networks, health literacy, out-of-hospital access to care, and prehospital delays in seeking care. We also lack data on the severity of the inciting stressor that precipitated the TTC event. This severity may vary systematically by geographic and socioeconomic context [19]. The absence of these measures means that residual confounding may remain despite our adjustment for measured covariates.

Our analysis is observational. Causality cannot be inferred from the observed associations [21]. While we adjusted for a broad set of measured confounders, we cannot rule out the possibility that unmeasured factors account for the differences we observed [22]. In particular, the NIS lacks granular clinical metrics such as baseline left ventricular ejection fraction, peak troponin levels, and the specific nature of the physical or emotional trigger (e.g., acute versus chronic, emotional versus physical). These variables are powerful drivers of TTC severity and could confound the associations between rurality, income, and mortality. The study period of 2022 represents a single year of data. This limits our ability to assess temporal trends or to validate findings in independent samples. The 2022 data may also reflect the ongoing effects of the COVID‑19 pandemic on healthcare delivery and outcomes. The magnitude and direction of any such effects are uncertain. The NIS does not capture out-of-hospital deaths or deaths occurring after discharge. Our analysis is limited to in-hospital mortality. If rural patients or low-income patients are more likely to die shortly after discharge due to differences in post-acute care or social support, our analysis would underestimate the full mortality burden of these disparities. We did not have data on whether hospitals had specific protocols for managing TTC. We also lacked data on the availability of advanced therapies such as mechanical circulatory support or the threshold for transferring patients to tertiary centers [15]. The generalizability of our findings may be limited by the fact that the NIS includes only community hospitals and excludes federal hospitals, such as Veterans Affairs facilities, and long-term acute care hospitals [16].

## Conclusions

In this nationally representative sample of adults hospitalized with TTC, both rural hospital settings and low socioeconomic status were independently associated with higher in-hospital mortality. These findings indicate that structural factors, including geographic access to specialized cardiovascular services and community-level economic resources, significantly influence outcomes in this stress-mediated condition. What sets this study apart is threefold. First, it is the first study to simultaneously examine the independent and combined effects of hospital rurality and community income level on TTC outcomes using a nationally representative database. Second, it demonstrates a graded, additive relationship where patients from rural, low-income communities experienced the highest mortality, lowest angiography utilization, and poorest discharge outcomes, revealing a double vulnerability. Third, we propose a novel pathophysiological framework linking chronic socioeconomic stress to TTC severity through allostatic load and sympathetic nervous system priming, a mechanism previously unexplored in the disparities literature. From a clinical standpoint, physicians should consider early transfer to catheterization-capable centers for rural patients and screen for socioeconomic barriers. From a societal perspective, policies strengthening rural healthcare infrastructure, expanding telecardiology services, and addressing social determinants of health are needed. Future research should validate these findings in clinical registries that capture left ventricular ejection fraction, troponin levels, and trigger specific data, and should test interventions targeting both geographic and economic vulnerability.
